# The Valuable Role of Imaging Modalities in the Diagnosis of the Uncommon Presentations of COVID-19: An Educative Case Series

**DOI:** 10.1155/2021/7213627

**Published:** 2021-10-13

**Authors:** Mohammadreza Khaleghi, Alireza Aziz-Ahari, Nahid Rezaeian, Sanaz Asadian, Amirsajjad Mounesi Sohi, Omid Motamedi, Shilan Azhdeh

**Affiliations:** ^1^Radiology Department, Iran University of Medical Sciences, Tehran, Iran; ^2^Rajaie Cardiovascular Medical and Research Center, Iran University of Medical Sciences, Tehran, Iran

## Abstract

The outbreak of coronavirus disease 2019 (COVID-19) in late 2019 rapidly turned into a global pandemic. Although the symptoms of COVID-19 are mainly respiratory ones, the infection is associated with a wide range of clinical signs and symptoms. The main imaging modality in COVID-19 is lung computed tomography (CT) scanning, but the diagnosis of the vast spectrum of complications needs the application of various imaging modalities. Owing to the novelty of the disease and its presentations, its complications—particularly uncommon ones—can be easily missed. In this study, we describe some uncommon presentations of COVID-19 diagnosed by various imaging modalities. The first case presented herein was a man with respiratory distress, who transpired to suffer from pneumothorax and pneumomediastinum in addition to the usual pneumonia of COVID-19. The second patient was a hospitalized COVID-19 case, whose clinical condition suddenly deteriorated with the development of abdominal symptoms diagnosed as mesenteric ischemia by abdominal CT angiography. The third patient was a case of cardiac involvement in the COVID-19 course, detected as myocarditis by cardiac magnetic resonance imaging (MRI). The fourth and fifth cases were COVID-19-associated encephalitis whose diagnoses were established by brain MRI. COVID-19 is a multisystem disorder with a wide range of complications such as pneumothorax, pneumomediastinum, mesenteric ischemia, myocarditis, and encephalitis. Prompt diagnosis with appropriate imaging modalities can lead to adequate treatment and better survival.

## 1. Background

Coronavirus disease 2019 (COVID-19) has become the major health issue of the decade. According to official statistics, COVID-19 has affected nearly 75 million people worldwide, causing more than 1.5 million deaths and leaving many with long-term disabilities (https://www.who.int/). It was first identified as a severe acute respiratory syndrome from an unknown cause, presenting with lower respiratory symptoms and pneumonia: dry coughs, fever, and shortness of breath [1]. The responsible viral pathogen, severe acute respiratory syndrome coronavirus 2 (SARS-CoV-2), seems to use its notorious spike proteins to bind with the receptors of angiotensin-converting enzyme 2 (ACE2), which is mostly expressed in pulmonary and cardiac cells, making the lungs and the heart the major targets for SARS-CoV-2 [2, 3].

Noncontrast lung computed tomography (CT) scanning remains the most accessible and readily available diagnostic test in the clinical setting, and the well-known imaging finding of bilateral peripheral patchy ground-glass opacification/consolidation pattern is characteristic [4, 5]. However, due to the vast and ambiguous clinical presentations of COVID-19, besides the lung CT, other imaging modalities should be drawn upon to elucidate the obscure COVID-19 presentations.

In this study, we describe several uncommon clinical presentations of COVID-19 and the use of imaging modalities in their diagnosis by explaining some atypical imaging findings in 5 patients in our referral hospitals. All 5 patients had a positive reverse transcription-polymerase chain reaction (RT-PCR) test, and other differential diagnoses were excluded by complementary exams.

Accordingly, the following is a description of 5 COVID-19 patients with uncommon manifestations of pneumothorax, pneumomediastinum, mesenteric ischemia, myocarditis, and encephalitis.

## 2. Case Presentations

### 2.1. Case 1: Pneumomediastinum and Pneumothorax

**Figure 1:**
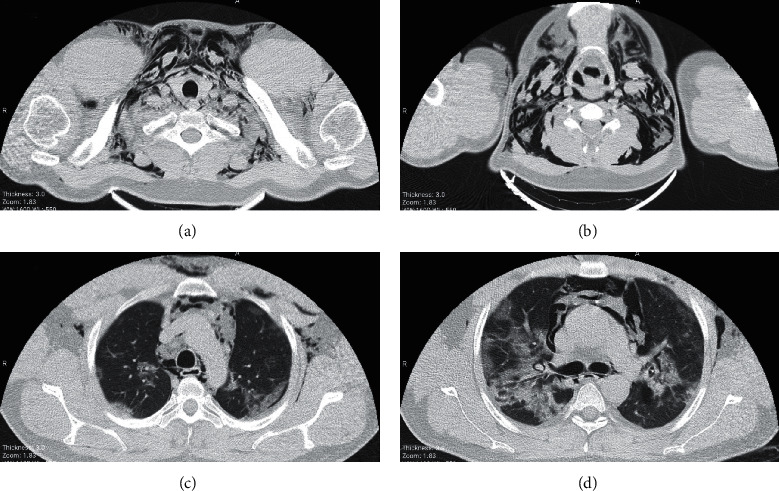
Axial images (a–d) of pulmonary computed tomography in the parenchymal window from the thoracic inlet and upper chest depict extensive pneumomediastinum, pneumothorax, and soft tissue emphysema. Note the peripheral patches of consolidation/ground-glass density.

**Figure 2:**
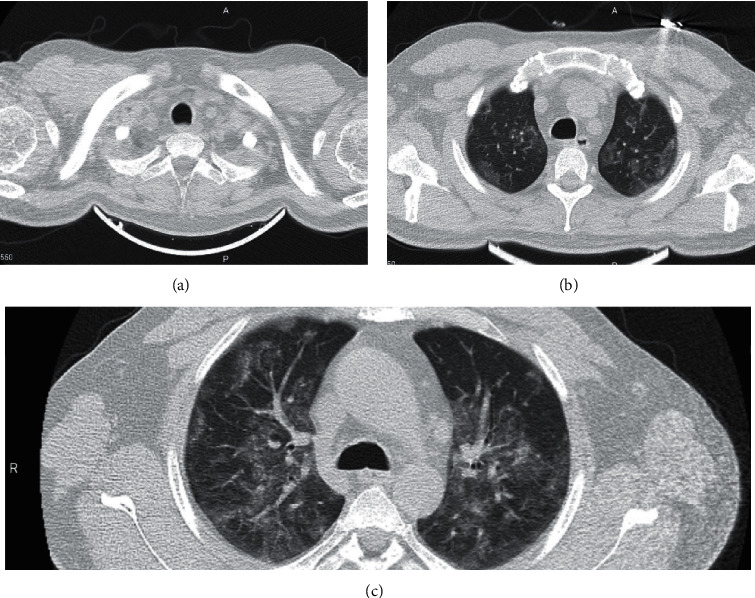
Axial images (a–c) of pulmonary computed tomography in the parenchymal window from the thoracic inlet and upper chest 2 months after the acute coronavirus infection. Complete resolution of the pneumomediastinum, pneumothorax, and soft tissue edema is evident. The persistence of the parenchymal opacities is predictable in all types of pneumonia including COVID-19-related ones.

A 35-year-old man with a negative past medical history was admitted to the emergency department with dyspnea, fever, and headaches of 6 days' duration. He had received azithromycin and dexamethasone as the treatment of pneumonia in another canter, but his symptoms had gradually worsened. An initial examination in our center revealed an oxygen saturation level of 88% in the room air, tachycardia (120 beats per minute), tachypnea (23 breaths per minute), and low-grade fever (∼38.3°C). The most prominent finding in the physical examination was bilateral subcutaneous emphysema in the neck with diminished respiratory sounds. Laboratory results showed elevated levels of 37 mm/h for the erythrocyte sedimentation rate (ESR) (normal range = 0–10) and 93 mg/dL for C-reactive protein (CRP) (normal range = 0–10). A blood count showed leukocytosis with 17.1 × 109 cells/L. Clinical suspicion of COVID-19 prompted PCR testing, which was positive for SARS-CoV-2. The patient underwent a lung CT scan, which showed characteristic ground-glass opacities, suggestive of COVID-19 in the peripheral subpleural regions of both lungs. Moreover, extensive free air was detected in the pleural and mediastinal cavities, compatible with the diagnosis of pneumothorax and pneumomediastinum (Figure 1). He was initially treated with oxygen supplementation with a reservoir mask; nonetheless, dyspnea exacerbation in addition to subcutaneous emphysema led to the intubation of the patient. A chest tube was inserted for the treatment of pneumothorax and pneumomediastinum. After 2 days, the patient was extubated and was given oxygen through a reservoir mask. His general condition improved during the admission. A CT scan on the eighth day of admission showed resolution of pneumothorax and pneumomediastinum and notable improvement of lung lesions (Figure 2). The patient was discharged after 9 days of hospital stay with an oxygen saturation level of 93% in the room air, white blood cell (WBC) count of 9.9 × 109 cells/L, ESR of 10 mm/h, and CRP of less than 6 mg/dL.

### 2.2. Case 2: Mesenteric Ischemia

A 54-year-old man with a 10-day history of worsening fever, coughs, and shortness of breath and without a remarkable past medical history was admitted. Initial examinations showed an oxygen saturation level of 84% in the room air, heart rate of 116 beats per minute, respiratory rate of 27 breaths per minute, and body temperature of 38.6°C. Laboratory evaluations showed leukocytosis with a WBC count of 13.9 × 10^9^ cells/L and elevated levels of ESR (91 mm/h) and CRP (27 mg/dL). With the clinical suspicion of COVID-19, the patient underwent a COVID-19 PCR test, which yielded a positive result. A lung CT scan demonstrated characteristic consolidations in the lower lobes of both the lungs with more severity on the left side, as well as ground-glass opacities in the subpleural areas.

**Figure 3:**
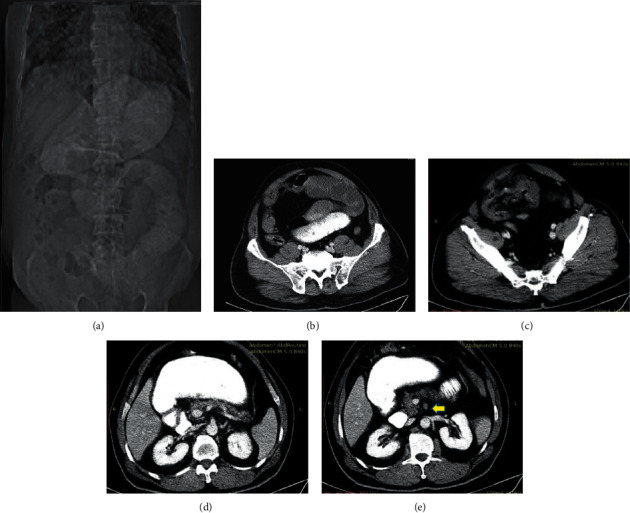
Abdominopelvic CT scan with an intravenous contrast agent. Topogram (a) and axial images (b, c) reveal mildly dilated hypoperistaltic bowel loops. (d, e) Axial images at the level of SMA origin and after separation of the inferior pancreaticoduodenal artery depict the normal origin of SMA with its dilatation, poor enhancement, and intraluminal linear filling defects (yellow arrow) in favor of thrombosis. SMA, superior mesenteric artery.

A combination of intravenous fluid therapy and hydroxychloroquine was initiated for the patient according to the institutional protocols. He also received oxygen supplementation via a nasal cannula. His condition gradually improved within 3 days of admission with favorable changes in the laboratory results: a return of the WBC count to the normal level and a relative decrease in inflammatory markers (ESR and CRP). Given the improvement, the patient was discharged. Two days later, however, he came to the emergency department complaining of suddenly developed periumbilical abdominal pain, nausea, and vomiting. A physical examination revealed abdominal distension and periumbilical tenderness. Electrocardiography was normal with sinus rhythm. An abdominal CT scan revealed nonmechanical bowel obstruction along with partial superior mesenteric artery thrombosis (Figure 3). The CT also showed that the origin of the superior mesenteric artery was normal, but it was occluded after the separation of the inferior pancreaticoduodenal artery. Upon mesenteric ischemia diagnosis, laparotomy was conducted. Small intestinal necrosis and discoloration of necrotic tissues were evident. Resection was performed on the necrotic segment (270 cm long: starting at 120 cm from the Treitz ligament and ending at 35 cm from the ileocecal valve) of the bowel, followed by an end-to-end anastomosis. A second-look surgery did not reveal any significant abnormality. The patient stayed in the intensive care unit for 3 days after the operation before he was transferred to the general ward. He was discharged from the hospital 3 days later with an acceptable general condition and the resolution of the presenting symptoms.

### 2.3. Case 3: Myocarditis

**Figure 4:**
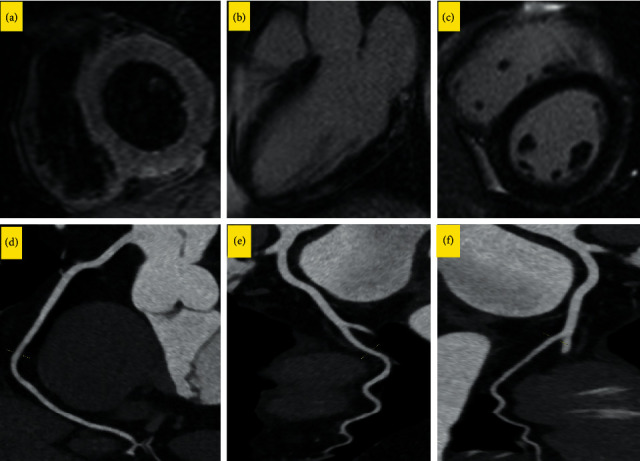
a–c) Cardiac magnetic resonance images. (a) Short-axis view short-tau inversion recovery sequence at the midventricular level demonstrates myocardial edema. (b, c) Three-chamber and short-axis LGE images depict midmyocardial-subepicardial fibrosis. (d–f) Curved multiplanar CT angiograms suggestive of normal epicardial coronary arteries.

A 36-year-old woman without a noteworthy past medical history was admitted to the hospital with dyspnea, fever, and tachycardia for the previous 5 days that had exacerbated. Vital signs on admission revealed an oxygen saturation level of 90% in the ambient air, tachycardia (126 beats per minute), a respiratory rate of 31 breaths per minute, and a body temperature of 38.4°C. A physical examination showed nothing remarkable except for diminished respiratory sounds in the bases of the lungs. The patient tested positive for COVID-19 PCR. A lung CT scan also confirmed the diagnosis with the visualization of peripheral ground-glass opacities, indicative of COVID-19. Laboratory results showed a normal count of WBC (9.17 × 10^9^/L) and elevated levels of ESR (32 mm/h) and CRP (21 mg/dL). Increased concentrations of lactate dehydrogenase (LDH) (1170 U/L, normal range = 135–225), creatine phosphokinase (563 U/L, normal range = 39–308), and troponin T (2991 pg/mL, normal range = 0–14) suggested the probability of myocardial injury. She was placed under electrocardiographic monitoring, which showed normal sinus tachycardia without evidence of ischemia. An echocardiographic examination revealed severe left ventricular dysfunction (ejection fraction = 25%). Subsequently, coronary CT angiography illustrated normal epicardial coronary arteries (Figure 4). A cardiac magnetic resonance imaging (MRI) revealed increased signal intensities in the myocardium in the short-tau inversion recovery sequence, consistent with myocardial edema. Furthermore, extensive subepicardial late gadolinium enhancement was detected (Figure 4). With the impression of COVID-19-related myocarditis, the treatment was started immediately with immunoglobulins, high-dose methylprednisolone, and remdesivir. By the sixth day of treatment, the cardiac function had improved notably on echocardiography (ejection fraction = 35%) and the oxygen saturation level had risen to 94%. The other vital signs were within normal ranges. The patient was discharged from the hospital 7 days after admission.

### 2.4. Case 4 and Case 5: Encephalitis

**Figure 5:**
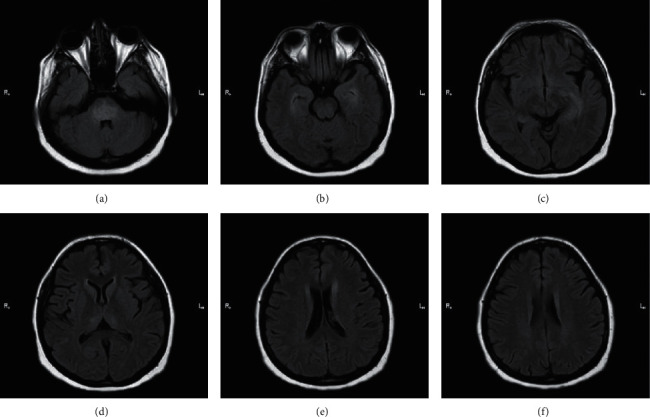
(a–f) Consecutive axial fluid attenuation inversion recovery sequence images of brain MRI without contrast. Increased signal intensity is evident in the pons (a), medial temporal structures (b, c), and thalami (d). Upper corticospinal pathways are spared (e, f).

A 36-year-old woman with a past medical history of type 2 diabetes mellitus was brought to the emergency department. On admission, the patient had severe shortness of breath, fever, fatigue, and weakness. The initial vital signs were an oxygen saturation level of 75% in the room air, pulse rate of 98 beats per minute, respiratory rate of 22 breaths per minute, and body temperature of 38.5°C. Laboratory data showed an elevated blood glucose level of 490 mg/dL, ESR of 40 mm/h, CRP of 40 mg/dL, a WBC count of 21.1 × 10^9^ cells/L, and a decreased blood pH level of 7.12. These findings were suggestive of diabetic ketoacidosis (DKA) in a known case of diabetes mellitus. Given the COVID-19 pandemic, decreased oxygen saturation, and fever, the patient was tested for COVID-19 with nasopharyngeal swab PCR testing, which yielded a positive result. A lung CT scan also confirmed the presence of bilateral ground-glass opacities and consolidations in the posterior segment of the upper lobes and the right lower lobe. She was intubated because of the exacerbation of dyspnea and the development of seizure. For the assessment of the infection of the central nervous system, a lumbar puncture was conducted. It showed a clear colorless sample with 4 WBCs/mm^3^ (normal range = 0–5), 80 RBCs/mm^3^ (normal range = 0), a glucose level of 80 mg/dL (normal range = 40–70), a protein level of 72 mg/dL (normal range = 15–45), and opening pressure of 16 H_2_O (normal range = 5–20). The cerebrospinal fluid (CSF) was negative for Gram staining, herpes simplex, cytomegalovirus, and the venereal disease research laboratory (VDRL) test. This CSF analysis raised the clinical suspicion of COVID-19-associated encephalitis or venous thrombosis. For further evaluation, brain MRI/magnetic resonance venography (MRV) was conducted. The brain MRI revealed increased signal intensity and volume in the bilateral medial temporal structures and thalami on the fluid-attenuated inversion recovery (FLAIR) sequence, with the increase being more prominent on the left side. Similar signal alterations were detected in the pons with the sparing of the corticospinal tracts (Figure 5). The brain MRV revealed no evidence of thrombosis in the dural venous sinuses. For the management of DKA, the patient received intravenous fluids and insulin for the reversal of the blood glucose level and the acidotic changes in arterial blood gases. The electrolytes were also controlled in the course of treatment. The patient came out of the DKA phase after 14 hours of treatment. For the treatment of COVID-19 and its associated encephalitis, she underwent therapy with methylprednisolone, intravenous immunoglobulins, and azithromycin. Her symptoms and consciousness ameliorated during the treatment. Two days after admission, the patient was extubated. On day 6 after admission, she had an oxygen saturation level of 96% without supplemental oxygen and normal pulse and respiratory rates. A CT scan on day 6 showed a decrease in the severity of pulmonary involvement. She was discharged from the hospital on the sixth day, with the recommendation of a repeat brain MRI after 1 month. Laboratory data on discharge demonstrated 12 × 10^9^ WBCs/L, ESR of 20 mm/h, and CRP of less than 6 mg/dL. The brain MRI in the following month showed no residual abnormalities or persistent lesions.

**Figure 6:**
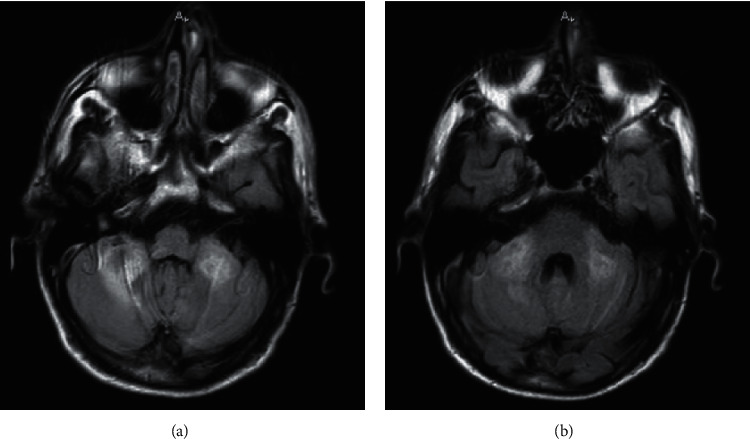
(a, b) Two consecutive axial fluid attenuation inversion recovery sequence images of brain MRI without contrast from the posterior fossa. Increased signal intensity is evident in bilateral cerebellar peduncles.

Our fifth case was a 52-year-old woman with a past medical history of hypertension. The patient was brought to the emergency department with the complaint of severe headaches, fatigue, dyspnea, and coughs. She had developed headaches and fatigue from 7 days earlier with gradual intensification. Two days before hospital admission, she developed dry coughs; on the day of admission, dyspnea. She had an oxygen saturation level of 90% in the room air, pulse rate of 92 beats per minute, respiratory rate of 18 breaths per minute, and body temperature of 37.8°C. Her SARS-CoV-2 PCR test was positive. Peripheral ground-glass opacities and diffuse consolidations with predominance in the lower lobes of both the lungs and lingula were detected. The severe headaches prompted an investigation for the involvement of the central nervous system. A lumbar puncture showed 2 WBCs/mm^3^, 150 RBCs/mm^3^, a protein level of 45 mg/dL, a glucose level of 60 mg/dL, negative Gram staining, no growth in bacterial culture, and negative results for all viral assays. The findings led to a diagnosis of possible COVID-19-associated encephalitis. Thereafter, a brain MRI showed increased FLAIR signal intensity in the bilateral middle cerebellar peduncles and cerebellar white matter, with the increase being more prominent on the right side (Figure 6). The treatment of encephalitis was mainly supportive, utilizing supplemental oxygen with a nasal cannula, remdesivir as antiviral therapy, and intravenous fluids for hydration. The encephalitis course was self-limiting with the resolution of the symptoms within 7 days. The patient was headache-free on the seventh day of admission with normal consciousness. Her respiratory symptoms decreased notably in comparison with the presentation day, and her oxygen saturation level increased to 94% in the room air. She underwent a follow-up lung CT scan, which showed the disappearance of the majority of the initial abnormal lesions on day 8 of admission. The patient was discharged with a good general condition and mild fatigue.

## 3. Discussion and Conclusions

COVID-19 has become the major health issue of the century in that it has affected the whole world. Primarily, COVID-19 manifested itself with lower respiratory symptoms such as coughs and dyspnea; nevertheless, the global spread of COVID-19 begot uncommon presentations [1]. We herein described 5 COVID-19 patients with rare and life-threatening complications, diagnosed with a timely application of imaging modalities. Pneumothorax and pneumomediastinum, mesenteric ischemia, myocarditis, and encephalitis are among the serious and uncommon complications of COVID-19, which should not be neglected and missed in COVID-19 patients with a rapid deterioration of clinical status.

Pneumothorax occurs in approximately 1% of all COVID-19 cases [6–8]. Although pneumothorax has been reported to be a consequence of barotrauma due to mechanical ventilation in acute respiratory distress syndrome, numerous studies have described the development of pneumothorax in patients on the nasal cannula or in ambient air [9]. Thus, spontaneous pneumothorax is now a well-documented rare complication of COVID-19, which chimes in with our presented cases. Pneumothorax might be a result of bilateral alveolar damage, leading to cystic changes. As the disease progresses, these cystic changes become more prone to rupture even in exposure to low pressures of nasal cannulae or coughs and other factors that increase transpulmonary pressures [10, 11]. In the radiologic survey of a patient with COVID-19, the formation of cystic changes and other contributing lesions to the development of pneumothorax must be assessed carefully. In addition to pneumothorax, there have also been reports of pneumomediastinum, which usually reflects more advanced lung involvement. In COVID-19 patients, pneumomediastinum is usually associated with more challenges in patient management and poorer outcomes [12]. Still, several case reports including ours have reported spontaneous resolution of this complication in patients with COVID-19 [13, 14]. Although the clinical behavior of pneumomediastinum is not clear in COVID-19 patients, radiologists must do their best for the early detection of findings related to the development of pneumomediastinum. A timely diagnosis of this condition determines the prognosis.

Thromboembolic events are a major group of complications in COVID-19. While deep venous thrombosis and pulmonary embolism carry a higher prevalence, acute mesenteric ischemia (AMI) is an overwhelming condition that necessitates early detection and management; otherwise, it will lead to catastrophic outcomes of death or severe morbidities [15]. The underlying mechanism of AMI in COVID-19 is not elucidated; however, several theories including hypercoagulable state due to inflammation, vascular thrombosis due to higher levels of von Willebrand factor in COVID-19 induced by endothelial damage, indirect intestinal damage by ACE2, and hemodynamic disturbances due to severe COVID-19 have been suggested [15–17]. Sudden occurrence of abdominal pain, nausea, vomiting, distension, or rapid deterioration of clinical status must prompt the clinical suspicion of AMI. Elevations in D-dimer and LDH levels may also be noted. On CT scans, dilation and edema in the bowel, intestinal wall thickening, pneumatosis intestinalis, and portal venous gas suggest the probability of AMI, which should be immediately explored with CT angiography as the gold standard for the detection of this devastating complication [17, 18]. Physicians must be alert about the signs and symptoms of AMI for the prevention of poor outcomes and higher survival of patients. Whenever there is a suspicion of AMI in COVID-19, workup and imaging must be instantly undertaken.

Cardiovascular complications of COVID-19 have been reported in between 7% and 31% of patients [19]. Myocarditis is a perplexing condition, and it is associated with 7% of COVID-19-related deaths [20]. Thus, the diagnosis and management of COVID-19 have a high clinical significance. Elevation of cardiac enzymes such as cardiac troponin I and NT-proBNP are the early clues for the investigation of myocarditis [21, 22]. While the endomyocardial biopsy is deemed the gold standard for the diagnosis of myocarditis [23], cardiac MRI is known as the gold-standard “imaging” modality for this condition [24, 25]. Different patterns of late gadolinium enhancement in COVID-19-associated myocarditis have been acknowledged in previous studies; nonetheless, it is mainly localized in the intramural or subepicardial regions of the left ventricle and the basal-to-mid inferolateral walls [22, 26].

Neurologic complications of COVID-19 comprise a large heterogeneous group of conditions. It is reported that 36% of hospitalized COVID-19 patients suffer from at least 1 neurologic complication [25]. Encephalitis is a known complication in many viral infections, and it causes major diagnostic challenges in COVID-19. A certain diagnosis of encephalitis is made through the isolation of the virus from the CSF. This process is usually not feasible in COVID-19 because of the transient dissemination of SARS-CoV-2 and its very low titers in the CSF [27]. Hence, brain MRI can be a useful tool in the diagnosis of COVID-19-associated encephalitis. Approximately half of COVID-19-encephalitis patients (51.5%) show no pathology in brain imaging. Of all the pathologies detected on brain MRI, diffuse hyperintensity of T2/FLAIR has been the most common, with its most prevalent location being in the white matter (24.2%), which is consistent with our findings [28]. An earlier study suggested a 41% rate for normal brain imaging in encephalitis [29]. Consequently, encephalitis in COVID-19 patients can be associated with nonspecific findings in brain MRI, electroencephalography, and the CSF analysis, rendering the role of physicians in ruling out the differential diagnoses and reaching the diagnosis of encephalitis critical. Several differential diagnoses can be suggested in the presence of seizure in COVID-19 patients with cerebral venous sinus thrombosis, which is a probable and dangerous condition due to the hypercoagulable state caused by viral infection. In the process of establishing the diagnosis of encephalitis, an appropriate choice of imaging modalities is crucial to differentiating encephalitis from venous thrombosis and other entities.

Our study describes uncommon and serious complications of COVID-19 that, if missed, could lead to mortality. We showed that clinical suspicion and choice of the right imaging modality could change the treatment strategy of patients and save lives in the multisystem disorder, that is, COVID-19, with a wide range of complications such as pneumothorax, pneumomediastinum, mesenteric ischemia, myocarditis, and encephalitis. Prompt diagnosis with appropriate imaging modalities can confer adequate treatment and improve survival.

## Data Availability

The data used to support the findings of this study are available from the corresponding author upon request.
